# Physical Activity Reduction and the Worsening of Gastrointestinal Health Status during the Second COVID-19 Home Confinement in Southern Italy

**DOI:** 10.3390/ijerph18189554

**Published:** 2021-09-10

**Authors:** Antonella Bianco, Isabella Franco, Alberto Rubén Osella, Gianluigi Giannelli, Giuseppe Riezzo, Caterina Bonfiglio, Laura Prospero, Paolo Sorino, Francesco Russo

**Affiliations:** 1 Laboratory of Epidemiology and Biostatistics, National Institute of Gastroenterology, “S. de Bellis” Research Hospital, 70013 Castellana Grotte, Italy; antonella.bianco@irccsdebellis.it (A.B.); isabella.franco@irccsdebellis.it (I.F.); arosella@irccsdebellis.it (A.R.O.); catia.bonfiglio@irccsdebellis.it (C.B.); paolosorino96@libero.it (P.S.); 2 Scientific Direction, National Institute of Gastroenterology, “S. de Bellis” Research Hospital, 70013 Castellana Grotte, Italy; gianluigi.giannelli@irccsdebellis.it; 3 Laboratory of Nutritional Pathophysiology, National Institute of Gastroenterology, “S. de Bellis” Research Hospital, 70013 Castellana Grotte, Italy; giuseppe.riezzo@irccsdebellis.it (G.R.); laura.prospero@irccsdebellis.it (L.P.)

**Keywords:** COVID-19, energy expenditure, gastrointestinal tract, GI symptoms, physical activity

## Abstract

Due to the COVID-19 pandemic, in December 2020, the Italian government established a second home confinement during the Christmas holidays. These restrictions offered the opportunity to utilize a well-defined model for observing the consequences of lifestyle changes of healthy individuals. This study aimed at estimating in healthy subjects from Southern Italy the physical activity (PA), the presence and the severity of gastrointestinal (GI) symptoms, and the association between the possible worsening of GI health status and the PA levels. An adapted version of the International PA Questionnaire-short form (IPAQ-SF) and the Gastrointestinal Symptom Rating Scale (GSRS) were proposed through Google’s online survey platform in three different periods via WhatsApp^TM^ to 499 healthy subjects (62% females) from Apulia (25%), Calabria (37%), and Campania (38%). Our results showed statistically significant changes during the home confinement: reduced energy expenditure (EE) among overweight subjects (−239.82, 95%CI −405.74; −73.89) or those who had high GSRS total score (−1079.54, 95%CI −2027.43; −131.66). An increase in GSRS total score was observed in overweight subjects, reaching statistical significance in those from Campania. Our study supports the importance of PA to reduce health risks, including those related to the possible onset of GI tract diseases.

## 1. Introduction

The pandemic due to Coronavirus Disease (COVID-19), caused by Severe Acute Respiratory Syndrome Coronavirus 2 (SARS-CoV-2), has led international and Italian authorities to adopt containment measures with drastic changes in social life and restriction in the movement of individuals [1].

These imposed constraints represented a unique and hopefully unrepeatable condition, offering the opportunity to utilize a well-defined model to observe and evaluate the consequences of the restrictions and changes in the lifestyle of healthy subjects.

In our previous study [2], performed during the first 2-month home confinement (March to May 2020), physical activity (PA) levels and energy expenditure (EE) (MET-minutes/week) were estimated in a cohort of subjects from the Southern Italian population before and during the home confinement. Evaluations were performed by administering online an adapted, validated, and already used version of the International Physical Activity Questionnaire-Short Form (IPAQ-SF) [3]. Results showed a statistically significant decrease in PA levels during the constraint period in both sexes, particularly in young subjects and adults [2]. As widely demonstrated in the literature, reducing daily EE and an increasingly sedentary lifestyle leads to an increased risk of developing several chronic diseases [4]. PA has been shown to reduce the risk and prevent a wide range of diseases, such as fibromyalgia, depression, hypertension, and diabetes mellitus [5,6,7], while also inducing positive effects on the gastrointestinal (GI) tract [8].

Increased PA has been proven to ameliorate symptoms in GI disorders, such as irritable bowel syndrome (IBS). Data in the literature show that physically active patients with IBS experience fewer symptoms than physically inactive ones [9]. This evidence overall suggests the possible use of the PA for managing IBS. The rationale of these positive effects relies on the fact that PA may augment intestinal microbial diversity through several mechanisms, including the promotion of an anti-inflammatory state [9,10].

During the Christmas season, to contrast the spread of the virus, the Italian government established an additional national holiday home confinement from 23 December 2020 to 7 January 2021 [11]. According to our previous results on the modifications of EE during the first home confinement period, it was conceivable that the second short home confinement could also have changed people’s PA levels. Additionally, given the close link between PA and the GI tract, this constraint period was likely to exacerbate GI health status. On these bases, the study was aimed at estimating in three cohorts of subjects practicing fitwalking from three Districts of Southern Italy: (a) the increased or decreased PA levels and their determinants; (b) the presence and the severity of GI symptoms identified by a validated GI questionnaire, the Gastrointestinal Symptom Rating Scale (GSRS), and (c) the association between the possible worsening of GI health status and the PA levels.

## 2. Materials and Methods

### 2.1. Study Design

The study was carried out in three different Italian Districts. Three Walking Fitness Associations were invited and accepted to participate: A.s.d. Apulia Fitwalking (Apulia), A.s.d. Atletica Salerno Gruppo Fitwalking (Campania), and A.s.d. Calabria Fitwalking (Calabria).

Associations were asked to distribute among their members via WhatsApp^TM^ the link to the Google online survey platform (Google L.L.C., Mountain View, CA, USA), where the data collection instruments were hosted. Participants completed the questionnaires three times in different periods: one week before 23 December 2020, at the end of the period between 23 December 2020 and 6 January 2021, and one month after the short home confinement. Participants <18 years old were not included in the study.

### 2.2. Data Collection

Questionnaires included an introductory page describing the background and the purpose of the study and the anonymity and confidentiality declarations.

### 2.3. Exposure Assessment

Participants completed the IPAQ-SF questionnaire [3]. The online self-reported questionnaire consisted of 31 items designed to measure frequency, duration, and intensity of the level of PA of participants.

To establish the EE of physical activities, we used the Metabolic Equivalent of Task (MET). It corresponds to 3.5 mL O_2_ kg^−1^ min^−1^ or 1 kcal kg^−1^ h^−1^. The weekly PA level was calculated as EE in MET*minutes*week-1 [12].

MET levels were obtained following the Compendium of Physical Activities (and subsequent updates) [13,14] and the Guidelines for Data Processing and Analysis of the International Physical Activity Questionnaire (IPAQ)—Short and Long Forms” [15]. The total weekly EE in MET*minutes*week-1 was estimated and categorized by applying the MET intensity values associated with walking (3.3), moderate (4.0), and vigorous (8.0) intensity physical activities [14,16]. The values obtained were successively categorized as Low, Moderate, and Vigorous levels of PA.

### 2.4. Data Privacy and Informed Consent

All participants were required to provide informed consent through an appropriate checkbox after being informed that all data would be used only for research purposes. Participants’ answers were anonymous and confidential, following Google’s privacy policy (https://policies.google.com/privacy?hl=it, Accessed on 2 January 2021). By completing the survey, they were asked to be as honest as possible in their responses [17]. Furthermore, the subjects’ participation was voluntary; they could spontaneously abandon the study at any time, and if they did so, the answers would not be saved.

### 2.5. Outcome Assessment

GSRS is a validated GI questionnaire that utilizes a 7-level Likert scale (1–7), based on the intensity and frequency of GI symptoms experienced during the previous seven days. A higher score represents the main symptoms complained about by the patients. The 7-level scores were then merged to obtain a 4-level score of intensity/frequency: absent, mild, moderate, and severe [18]. In order to obtain a unique index representing the global health GI status, we calculated the GSRS total score in all the healthy subjects practicing fitwalking. Then, this value was analyzed in relation to other indexes evaluated (e.g., EE) before, during, and after the constraint period.

### 2.6. Statistical Analysis

Data description was performed using means (SD) and frequencies (%) and compared with ANOVA or Chi-squared test as appropriate. 

The statistical analysis was carried out in three steps. Firstly, we fitted a model with EE (MET*min*week-1, continuous) as a dependent variable to probe the relationship between the covariates and the outcome. Secondly, to explore the effect of PA intensity (walking, moderate, and vigorous), a second model was fitted with GSRS total score (continuous) as a dependent variable adjusted by several covariates. Finally, we categorized the GSRS total score and used the moderate and severe categories to probe the relationship between the covariates and the outcome.

A Generalized Estimating Equation (GEE) [19] was performed to evaluate the effect of the covariates on EE or GSRS total score (continuous or categorized). GEE models are helpful in biomedical studies to estimate how the average outcome changes with covariates, allowing correlated response data (repeated measurements on each subject). A gamma (link identity) or binomial (link logit) distribution for the dependent variables was assumed for continuous (MET*min*week-1) and categorical (GSRS total score) variables, respectively. An unstructured correlation matrix was set to the data. Gender (categorical), Age (continuous), BMI (categorical: <25, 25–29.9 and ≥30), District (Apulia, Calabria, and Campania), Time (Before, During, and After home confinement), a modification effect term between exposure (GSRS total score and EE level, as appropriate), and Time spent while sitting were included as covariates. Several effect modification terms between District, Sex, BMI, EE level, and GSRS total score were probed. Moreover, by using post-estimation options, several contrasts between time (Before, During, After) and GSRS categories (Absent, Mild, Moderate, and Severe) were tested. The results obtained are expressed in natural scale as mean or Odds Ratio (OR) ±95% Confidence Interval (95% CI) for continuous or categorical variables. A Wald test was applied to check the equality of coefficients between any pairwise comparisons of time and GSRS total score (on MET*minutes*week-1) or Level of EE (on GSRS total score continuous or categorized). All statistical analyses were performed using Stata 16.1 (StataCorp, 4905 Lakeway Drive, College Station, TX 77845, USA). In particular, the user-written program -qic- was used to determine the best correlation structure and -margins- and -marginsplot- to find out expected values.

## 3. Results

### 3.1. Participants Description

Participants’ characteristics are shown in Table 1. Four hundred and ninety-nine (62% females) out of 501 subjects were included. Two subjects were excluded because they were under 18 years old. At baseline, 88 (17.6%), 217 (43.7%) and 194 (38.7%) had Low, Medium, and High EE, respectively. Participants were fairly evenly distributed among regions: 126 from Apulia (25%), 188 from Campania (38%), and 185 from Calabria (37%). The mean age was 49.26 (±11.71). Eighty-two percent of participants were adults between 35 and 64 years old. As expected, most participants had a medium or high EE and were normal or overweight at BMI. Participants with a High Level of EE were less likely to spend time sitting before the home confinement (*p* = 0.03). The mean of GSRS total score was lower among participants with a High level of EE Before (22.57) and After home confinement (21.99) than Medium or Low EE levels (*p* = 0.003 and 0.006, respectively). There were no differences in GSRS total scores before and during the home confinement. However, those subjects with high levels of EE were less likely to exhibit Severe symptoms (*p* < 0.001).

When the GSRS score was considered in terms of Absent/Mild vs. Moderate/Severe, it was observed that before home confinement, about 46% of participants who had Medium or High EE were concentrated in the Absent/Mild category, whereas only 36% of those who had Moderate/Severe symptoms belonged to the same EE category (*p* < 0.02). During the home confinement, participants with Absent/Mild (22.4%) and Moderate Severe (32.3%) symptoms had a Medium EE without reaching statistical significance. After the home confinement percentages of High EE returned to the level of Before home confinement (*p* < 0.01) with a greater increase in the Absent/Mild category than the pre home confinement level (Table 2).

### 3.2. Determinants of EE Decreasing during the Home Confinement

**Figure 1:**
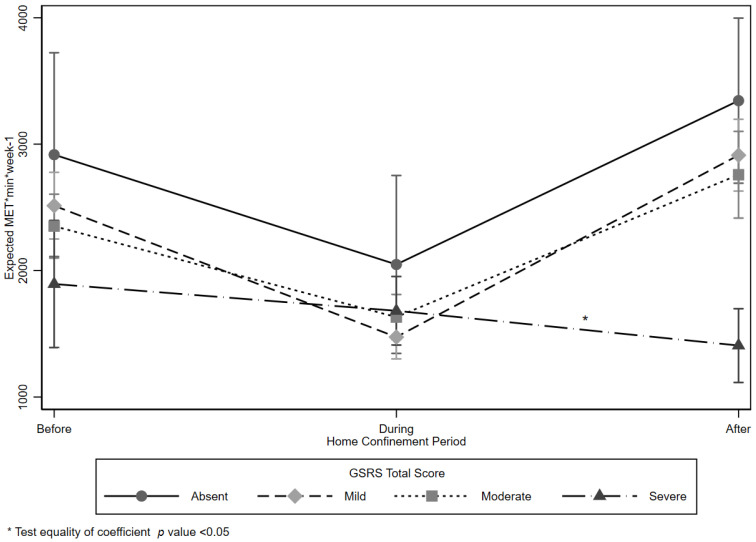
Expected energy expenditure expressed as MET*min*week-1 by Gastrointestinal Symptom Rating Scale (GSRS) total score and Time.

Results from the GEE analysis are shown in Table 3. There was no effect of District and Time on EE, and there was no modification effect between time and GSRS total score. There was a statistically significant reduction of MET*min*week-1 among persons who were overweight (−239.82, 95%CI −405.74; −73.89), spent more time sitting (−0.68, 95%CI −1.12; −0.23), or had a high GSRS total score (−1079.54, 95%CI −2027.43; −131.66). Men were more likely to have a higher EE than females (316.71, 95%CI 143.16; 490.28). Contrasts between time and GSRS categories are also shown in Table 3 and graphically displayed in Figure 1.

### 3.3. Determinants of GSRS Total Score Changes during Home Confinement

**Figure 2:**
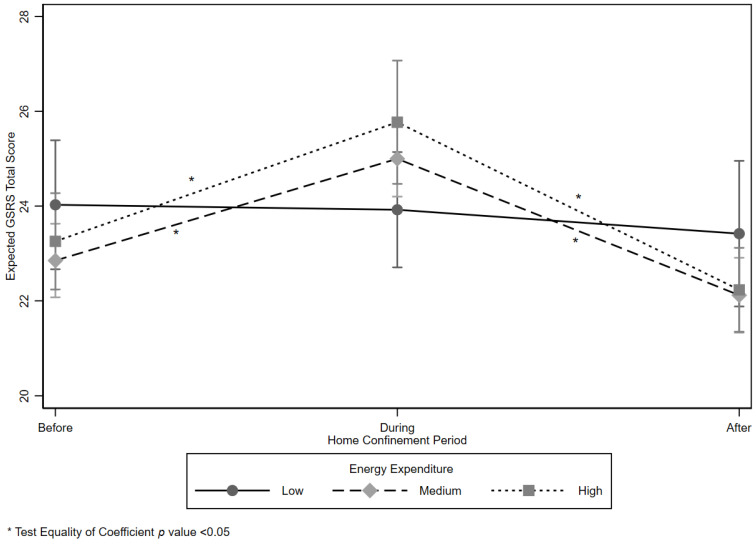
Expected mean Gastrointestinal Symptom Rating Scale (GSRS) total score by Energy Expenditure (EE) level and time.

Results from the GEE analysis are shown in Table 4. There was a positive statistically significant effect among participants coming from the Campania District (1.31, 95% CI 0.58; 2.05). A negative statistically significant effect was evident among males (−1.55, 95% CI −2.15; −0.96), among those who spent more time sitting (−0.002, 95% CI −0.004; 0.001). Although there were two main negative statistically significant effects among subjects who had a Medium (−1.47, 95% CI −2.91; −0.37) or High (−1.99, 95% CI −3.49; −0.54) EE, these effects were positively statistically significant when considered in a modification effect term of time*EE (During*Medium 2.14, 95% CI 0.24; 40.5, and During*High 2.93, 95% CI 0.78; 4.05). Expected probabilities of GSRS total scores by level of EE and Time are graphically displayed in Figure 2 and the test of equality of coefficients.

### 3.4. Symptoms Severity and Some Potential Determinants

**Figure 3:**
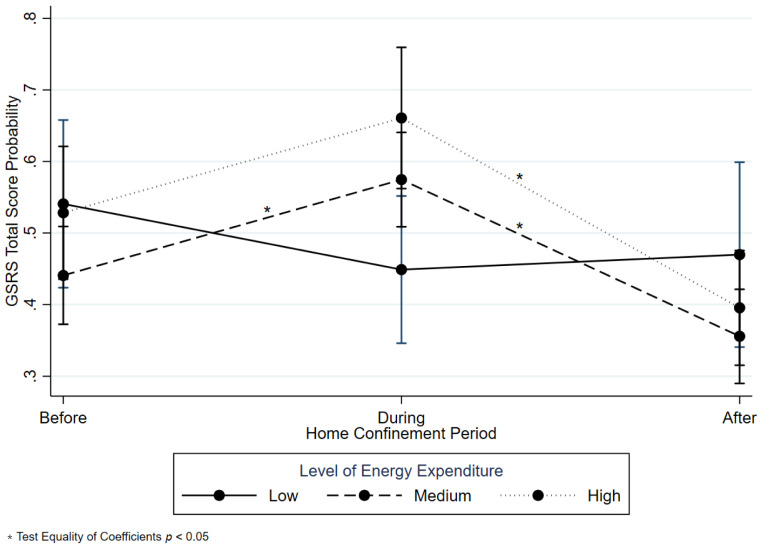
Generalized Estimating Equation. Gastrointestinal Symptom Rating Scale (GSRS) total score probabilities by the level of EE and Time.

Results from the GEE analysis are shown in Table 4. By comparing Medium and Severe with Absent and Mild GSRS total score, a negatively statistically significant association among participants from the Calabria District (OR 0.63, 95% CI 0.48; 0.82), males (OR 0.61, 95% CI 0.49; 0.76), and Medium (OR 0.57, 95% CI 0.34; 0.96) and High EE (OR 0.57, 95% CI 0.34; 0.96) was observed. A positively statistically significant association was found between overweight subjects coming from the Campania District (OR 1.31, 95% CI 1.00; 1.72) and overweight participants (OR 1.34, 95% CI 1.07; 1.67). A modification effect was found between Time and EE level (During*Medium OR 2.52, 95% CI 1.29; 4.93 and During*High OR 2.96, 95%CI 1.38; 6.34). Expected probabilities of GSRS severity scores by level of EE and Time are graphically displayed in Figure 3 together with the test of equality of coefficients.

## 4. Discussion

This panel study showed several statistically significant changes in mean EE and the worsening of GI health status as measured by the GSRS total score

PA plays a fundamental role in the management and prevention of many diseases, including chronic and autoimmune diseases [20,21]. Regular PA and exercise are associated with numerous physical and mental health benefits in men and women, and it delays all-cause mortality, as widely highlighted in the literature [22]. A physically active lifestyle decreases the risk of developing coronary heart disease, stroke, type 2 diabetes, and some forms of cancer (e.g., colon and breast cancers) [23]. Furthermore, daily moderate PA reduces the risk for some GI disorders and the gravity of chronic GI symptoms [8]. Additionally, several studies have demonstrated the positive PA effects and recommended exercise as a treatment option in patients with IBS [9,10].

Our bodies need a relatively long period to benefit from the healthy adaptations that exercise and PA can generate, modulated by different mechanisms, such as epigenetic factors, metabolic pathways, or reduced inflammation [24,25,26,27]. However, it requires only a few days to reverse these adaptations, and the body returns to a physiological situation similar to baseline or even worse [28]. So, the Christmas home confinement seemed to be a sufficient period to trigger changes in our cohorts of subjects.

Firstly, the current results show that METs increased on average in men but not in women, according to previous reports on higher male PA and EE [2,29]. Secondly, METs decreased in overweight subjects but not in normal-weight or obese people. It is known that obese subjects are less likely to carry out PA Inactivity and sedentary lifestyle are some of the causes of obesity [30]; therefore, they probably did not do exercise before the COVID-19 home confinement and remained inactive during and after the home confinement. Remarkably, the METS decreased in subjects with severe GSRS symptoms. This reduction could be attributed to different reasons, such as stress factors [31,32,33,34], new conditions imposed by the constraint period, overtraining, or incorrect training [8,35]. Due to these conditions, the possible drop-out can explain the continuous reduction of METs, even in the observed post-home confinement period. However, in this context, the reverse causation bias cannot be ruled out [36].

As concerns PA levels in relation to GI health status, our data support the positive effects of exercise on the GI tract [9]. These benefits were more marked in men than women. The present finding was not surprising since, as already reported, men generally appear to be more active than their female counterparts [2,29]. 

Additionally, the decrease in the GSRS total score was more marked among subjects who claimed to perform high and medium PA than those performing low PA. In this regard, other studies have previously reported that PA in general and vigorous activity, in particular, protect from GI disorders, such as the onset of diverticular disease of the colon [37]. Therefore, high and medium PA could also positively affect subjects with GI health status. However, the increased GSRS total score observed among participants coming from the Campania District remains unexplained as we do not have sufficient evidence that this result may be due to particular lifestyle patterns (e.g., eating habits, drinking, or PA). Another intriguing result is the increase in GSRS total score in Before vs. During home confinement time, but we could not find similar results in the literature.

GSRS total score categorized analysis found that overweight but not obese subjects were more likely to present severe symptoms. Several studies have determined that obesity is a clear potential risk factor for various diseases and that approximately 50% of patients with cancer had an abnormally high BMI [38,39]. So, we should have found greater significance in obese rather than overweight subjects. Nevertheless, generally, self-reported surveys are strongly correlated with social desirability bias, so we have to consider the reliability of the responses to our questionnaires [40]. The association between GSRS total score and District remains unexplained for us.

Even if the sample size is relatively large and our estimates are reliable, several issues need to be considered. First of all, as data on this topic are still insufficient, we cannot compare our results with data in the literature. The present study also shows some limitations, such as the lack of information about participants’ lifestyles, especially health status and eating habits, and the short duration of the observation. As mentioned above, the socially desirable answer bias may be present in our study, meaning our estimates may be biased toward the null hypothesis.

## 5. Conclusions

In conclusion, despite being a short period, the Christmas home confinement led to a significant reduction in EE and PA levels and increased GI symptoms, especially in overweight subjects. This negative effect of home confinement is undoubtedly due to restrictions and confinement. One month after the short home confinement, once the possibility of walking outdoors was regained, our results showed increased EE and an improvement of the GI health status. In the absence of data regarding other factors that may have influenced our results (e.g., eating habits and mental well-being), we can assert that according to the major institutions, namely, the World Health Organization [41] and the American College of Sports Medicine [42], increasing PA is recommended to reduce health risks, premature mortality, and numerous chronic medical conditions [43,44,45,46]. Several studies have reported the importance of regular PA and exercise for the primary and secondary prevention of many chronic medical conditions [47], and we can affirm, as also supported by our present results, the GI tract as well [8,9,10].

## Figures and Tables

**Table 1:** Characteristics of Participants Before, During, and After Home confinement.

Variable	Energy Expenditure			
	Low	Medium	High	*p*-Value *
**Age** (years)	49.35 (10,11)	49.03 (11,11)	49.48 (13,02)	0.92
**Age** (categorized)				
18–34	8 (14.3%)	23 (41.1%)	25 (44.6%)	0.74
35–64	74 (18.1%)	181 (44.4%)	153 (37.5%)	
≥65	6 (17.1%)	13 (37.1%)	16 (45.7%)	
**Sex**				
Female	59 (19.0%)	135 (43.4%)	117 (37.6%)	0.56
Male	29 (15.4%)	82 (43.6%)	77 (41.0%)	
**District**				
Apulia	17 (13.5%)	64 (50.8%)	45 (35.7%)	0.18
Calabria	30 (16.2%)	80 (43.2%)	75 (40.5%)	
Campania	41 (21.8%)	73 (38.8%)	74 (39.4%)	
**BMI** (categorized)				
<25	38 (14.3%)	112 (42.3%)	115 (43.4%)	0.096
25–30	39 (20.6%)	83 (43.9%)	67 (35.4%)	
>30	11 (24.4%)	22 (48.9%)	12 (26.7%)	
**Time spent sitting** (min)				
Before	205.18 (148.84)	223.38 (130.99)	190.10 (119.04)	0.034
During	424.31 (172.37)	383.05 (165.45)	410.63 (200.15)	0.066
After	227.19 (153.40)	250.11 (150.23)	222.54 (133.96)	0.13
**GSRS total score**				
Before	24.73 (5.50)	22.92 (5.04)	22.57 (4.86)	0.003
During	24.83 (6.36)	25.16 (6.50)	25.36 (6.60)	0.82
After	24.63 (6.20)	22.46 (5.89)	21.99 (5.69)	0.006
**GSRS** (categorized)				
**Before**				
Absent	5 (15.6%)	11 (34.4%)	16 (50.0%)	0.092
Mild	30 (12.9%)	109 (47.0%)	93 (40.1%)	
Moderate	45 (22.1%)	82 (40.2%)	77 (37.7%)	
Severe	8 (25.8%)	15 (48.4%)	8 (25.8%)	
**During**				
Absent	3 (15.8%)	10 (52.6%)	6 (31.6%)	0.41
Mild	57 (30.2%)	102 (54.0%)	30 (15.9%)	
Moderate	53 (25.4%)	113 (54.1%)	43 (20.6%)	
Severe	17 (20.7%)	48 (58.5%)	17 (20.7%)	
**After**				
Absent	4 (7.3%)	23 (41.8%)	28 (50.9%)	<0.001
Mild	22 (9.1%)	92 (38.0%)	128 (52.9%)	
Moderate	20 (13.0%)	53 (34.4%)	81 (52.6%)	
Severe	16 (33.3%)	21 (43.8%)	11 (22.9%)	

* ANOVA for continuous and X^2^ for categorical variables were applied where appropriate. Cell content: mean (SD) or frequency (%) as appropriate. BMI: Body Mass Index; GSRS: Gastrointestinal Symptom Rating Scale.

**Table 2:** Distribution of Participants by Time, Energy Expenditure (MET*min*week-1) and Categories of GSRS Severity.

Variable	Energy Expenditure			
	Low	Medium	High	*p*-Value *
**GSRS** (categorized)				
**Before**				
Absent/Mild	35 (7.0%)	120 (24.0%)	109 (22.0%)	0.024
Moderate/Severe	53 (11%)	97 (19.0%)	85 (17.0%)	
**During**				
Absent/Mild	60 (12.0%)	112 (22.4%)	36 (7.3%)	0.40
Moderate/Severe	70 (14.0%)	161 (32.3%)	60 (12.0%)	
**After**				
Absent/Mild	26 (5.3%)	115 (23.0%)	156 (31.3%)	0.010
Moderate/Severe	36 (7.2%)	74 (14.8%)	92 (18.4%)	

* X^2^ for categorical variables; Cell content: frequency (%); GSRS: Gastrointestinal Symptom Rating Scale.

**Table 3:** Generalized Estimating Equation. Determinants of Energy Expenditure (EE) expressed as MET*min*week-1 during the home confinement.

Variable		
	Coeff ^†^	95% CI ^‡^
**Sex**	316.7 **	143.1; 490.3
**BMI** (categorized)		
<25	0	
25–30	−239.8 *	−405.7; −73.9
>30	−148.7	−438.7; 141.3
**District**		
Apulia	0	
Calabria	162.4	−50.9; 375.7
Campania	−115.2	−317.3; 86.9
**Time spent sitting**		
Before	0	
During	−881.3	−1962.9; 200.2
After	348.8	−692.8; 1390.4
**GSRS** (categorized)		
Absent	0	
Mild	−426.1	−1271.3; 419.2
Moderate	−574.2	−1417.4; 269.1
Severe	−1079.5 *	−2027.4; −131.6
**Time#GSRS total score**		
(During vs. Before) (1 vs. 0)	−152.4	−1269.7; 964.9
(During vs. Before) (2 vs. 0)	172.0	−947.2; 1291.3
(During vs. Before) (3 vs. 0)	722.9	−495.9; 1941.7
(After vs. During) (1 vs. 0)	29.2	−1080.6; 1139.0
(After vs. During) (2 vs. 0)	121.6	−1004.7; 1247.8
(After vs. During) (3 vs. 0)	−834.1	−2025.7; 357.5
**Sitting minutes**	−0.67 *	−1.12; −0.23

^†^ Generalized Estimating Equation, age-adjusted estimates; ‡ 95% Confidence Interval; * *p*-value < 0.05; ** *p*-value < 0.001. BMI: Body Mass Index; GSRS: Gastrointestinal Symptom Rating Scale.

**Table 4:** Generalized Estimating Equation. Determinants of GSRS total score changes during the home confinement.

Variable	GSRS		GSRS (Absent/Mild vs. Medium/Severe)	
	Coeff ^†^	95% CI ^‡^	OR	95% CI ^‡^
**Sex**	−1.55 **	−2.15; −0.95	0.61 **	0.49; 0.76
**BMI** (categorized)				
<25	0		1	
25–30	0.58	−0.03; 1.19	1.34 *	1.07; 1.67
>30	−0.55	−1.54; 0.45	1.15	0.79; 1.69
**District**				
Apulia	0		1	
Calabria	−0.36	−1.07; 0.36	0.63 **	0.48; 0.82
Campania	1.31 **	0.58; 2.05	1.31 *	1.01; 1.71
**Time**				
Before	0		1	
During	0.39	−1.27; 2.06	0.85	0.47; 1.52
After	−0.19	−2.10; 1.71	0.88	0.45; 1.71
**Energy Expenditure**				
Low	0		1	
Medium	−1.47 *	−2.91; −0.04	0.57 *	0.34; 0.96
High	−1.99 *	−3.44; −0.54	0.57 *	0.33; 0.96
**Time#Energy Expenditure**				
(During vs. Before) (Medium vs. Low)	2.14 *	0.24; 4.05	2.52 *	1.28; 4.93
(During vs. Before) (High vs. Medium)	2.93 *	0.78; 5.08	2.96 *	1.38; 6.34
(After vs. During) (Medium vs. Low)	−0.30	−2.48; 1.87	0.92	0.42; 2.01
(After vs. During) (High vs. Medium)	−0.37	−2.52; 1.78	0.85	0.39; 1.84
Sitting minutes	−0.002 *	−0.004; −0.000	1.00	0.99; 1.00

^†^ Generalized Estimating Equation, age-adjusted estimates; ^‡^ 95% Confidence Interval; * *p* value < 0.05. ** *p*-value < 0.001. BMI: Body Mass Index. GSRS: Gastrointestinal Symptom Rating Scale.

## Data Availability

The datasets used and analyzed during the current study are available from the corresponding author on reasonable request.
